# The "Peculiar People"

**Published:** 1897-11-06

**Authors:** 


The Hospital

A Journal of

The Medical Sciences and Hospital Administration.

Vol. XXIII.—No. 580.] [Nov. 6, 1897.

### The "Peculiar People."

The conviction for manslaughter of Thomas George Senior and George Viner before Mr. Justice Ridley in the Central Criminal Court on Thursday in last week, and the somewhat lame and impotent conclusion to which that conviction was brought by the sentence delivered on the following day, seem to call for observation. The prisoners belong to the sect known as the "Peculiar People," a sect, the members of which, in the words of the Judge, arrogate to themselves that they alone, among all races of the earth, rightly understand the Word of God. The main article of their faith is that it is impious, according to the Scriptures, to call in medical aid; and the prisoners had refused to obtain such aid for their children, who died, if not for the want, yet certainly in the absence of it. Both the prisoners were said to be respectable men, leading creditable lives in regular employment, and to be in all respects good citizens, except in their disobedience to the law which enjoins upon a parent the duty of procuring a doctor for a sick child. The prisoner Senior had been the father of eleven children, six of whom had died in circumstances similar to those disclosed at the trial, and neither he nor Viner pleaded either ignorance of the law or want of power to comply with its requirements, but simply the pressure of what they conceived to be a higher duty. These sectaries are understood to take their stand upon two verses in the last chapter of the Epistle of St. James, which run as follows:—

"Is any sick among you? Let him call for the elders of the church; and let them pray over him, anointing him with oil in the name of the Lord."

"And the prayer of faith shall save the sick, and the Lord shall raise him up; and if he have committed sins, they shall be forgiven him."

It is, of course, difficult to reason with persons who are possessed by a fixed idea arising out of their conception of religious duty; but it would be kind, on the part of any persons in authority who were brought into contact with these poor deluded people, at least to try and convince them that the Scripture, in many places, declares compliance with the law to be a plain duty; that the Act of Parliament of 1868 expressly directs parents to procure medical assistance for sick children; that the Court of Crown Cases has on two occasions declared that the parent could not be exempted from this duty on the ground of his religious opinions; that medical advice, in the modern sense, was not to be procured when St. James wrote, and would almost certainly have been approved of by him, if he had foreseen what it was to become; and lastly, as already pointed out, that the words contain no prohibition. We believe that members of the sect have already so far departed from their original position as to admit doctors in cases of infectious illness; and it would seem to be the part alike of wisdom and of humanity to endeavour to furnish them with a way of escape from an utterly untenable position.

At the same time, while fully sympathising with Mr. Justice Ridley's desire to avoid making martyrs of these unfortunate people, we must confess to the opinion that this desire was permitted to influence him in an undue degree. The law has made it a crime to neglect to furnish sick children with medical attendance, and the prisoners were found guilty of manslaughter; yet they were only ordered to enter into their own recognizances in the sum of £20 to come up for judgment if called upon, the Judge warning them that, if they repeated the offence of which they had been convicted, they certainly would be called upon, and would, he hoped, be sentenced in a manner commensurate with what they persisted in doing. This might have been very well if the case had been the first of its kind, or if the offence had been committed for the first time by the prisoners. In the case of Viner, this seems to have been the fact; but Senior had already lost six children, and, in any other department of criminal jurisprudence, would have been regarded as a hardened offender. We do not see that it is compatible with the duty of a judge practically to withhold the protection of the law from certain children, merely because their parents entertain a particular religious belief, which, moreover, the Court of Appeal has already decided to furnish no answer to the offence. It is not so much the scruples of the parents as the safety of their remaining children that should be considered; and it is impossible to doubt that this safety has been seriously imperilled by the proceedings of last

Friday. Although convicted by the jury the prisoners have been virtually acquitted by the judge; and others in the same position will be likely to be encouraged in their folly, and to look for similar indulgence. We think that the dead child should have been made to outweigh the living scruple, and that a few sharp sentences would be likely to assist the Peculiar People generally in taking a more reasonable view of the nature and limitations of the apostolic injunction.

